# A Novel Method for Estimating Monocular Depth Using Cycle GAN and Segmentation

**DOI:** 10.3390/s20092567

**Published:** 2020-04-30

**Authors:** Dong-hoon Kwak, Seung-ho Lee

**Affiliations:** 1 Department of Electronic Engineering, Hanbat National University, Daejeon 34158, Korea; dsy0007@naver.com; 2 Department of Electronics & Control Engineering, Hanbat National University, Daejeon 34158, Korea

**Keywords:** monocular depth estimation, cycle GAN, segmentation, multi-task learning, adversarial loss, cycle consistency loss

## Abstract

Modern image processing techniques use three-dimensional (3D) images, which contain spatial information such as depth and scale, in addition to visual information. These images are indispensable in virtual reality, augmented reality (AR), and autonomous driving applications. We propose a novel method to estimate monocular depth using a cycle generative adversarial network (GAN) and segmentation. In this paper, we propose a method for estimating depth information by combining segmentation. It uses three processes: segmentation and depth estimation, adversarial loss calculations, and cycle consistency loss calculations. The cycle consistency loss calculation process evaluates the similarity of two images when they are restored to their original forms after being estimated separately from two adversarial losses. To evaluate the objective reliability of the proposed method, we compared our proposed method with other monocular depth estimation (MDE) methods using the NYU Depth Dataset V2. Our results show that the benchmark value for our proposed method is better than other methods. Therefore, we demonstrated that our proposed method is more efficient in determining depth estimation.

## 1. Introduction

In the field of image processing, three-dimensional (3D) images contain spatial information such as depth and scale, in addition to visual information. This 3D information is indispensable for virtual reality, augmented reality (AR), and autonomous driving. Modern society is currently undergoing a ‘Fourth Industrial Revolution’, which requires that technologies acquire and calculate 3D information quickly and accurately. To obtain this 3D information, radar, ultrasonic, and laser sensors have been developed, and 3D photography methods utilizing specialized cameras such as stereo cameras have been proposed [1,2].

Recently, research on obtaining 3D information from single images using machine learning has been increasing. Compared with existing methods, which require specialized equipment and sensors, our new method can generate 3D information using a single camera. This has advantages in terms of low cost, high scalability, easy miniaturization, and ease in obtaining single image data. Moreover, estimating depth information in stereo images is important, especially for applications such as Optical Flow and Point Cloud, where other information cannot be used. We, therefore, propose a method for improving the precision of depth by converting image data into two forms of segmentation and depth using backpropagation of the cycle consistency loss of cycle generative adversarial network (GAN). Cycle GAN was used because it supports relatively high resolutions, compared with other networks. Although the amount of data required for learning is large, the shape of the RGB image data does not change. Our proposed method only uses a single RGB image to estimate depth information. The image data are then converted to depth information while still maintaining the characteristics of the RGB image through consistency loss of cycle GAN. In addition, our method minimizes the loss of depth information by adding consistency loss through segmentation. It also resolves the fading problem, where depth information appears ambiguous or is hidden by larger features. Our proposed method, therefore, estimates the depth information of single images using cycle GAN and can be used for fast and precise 3D transformation of 3D computer graphics, as well augmented reality (AR) and autonomous driving, which require 3D information.

## 2. Literature Review

### 2.1. Depth Estimation Methods Before GAN

Over the past few years, depth information from images has largely been obtained from stereo images or video sequences. In 2014, Zbontar et al. [3] proposed a depth estimation method for stereo images using convolutional neural network (CNN) [4] and depth estimation methods based on stereo images using a fully convolutional network [5], while recurrent neural network [6] and other similar technologies have been since proposed. Furthermore, Fanello et al. [7] proposed an algorithm for determining a correlation between pixels and absolute depth measurements. Using this algorithm, 3D images of human hands and faces were created by estimating the depth information of the hands and faces using only smartphone cameras. Since then, studies on enhancing the quality of depth information have been readily conducted. Ha et al. [8] estimated that high-quality depth information could be obtained through self-calibration bundles adjusted in line with small motions. This contrasts with conventional methods that restore images by correcting for geometric characteristics and motions of cameras in advance. Kong et al. [9] integrated the depth, albedo, shading, optical flow, and surface contours of the images to obtain more precise depth estimations by utilizing several images and mathematical calculations. 

### 2.2. Monocular Depth Estimation Methods

Depth information cannot be calculated using the aforementioned methods in monocular depth estimation (MDE), where 3D information is estimated from individual images. Therefore, machine learning is required. Eigen et al. [10] estimated depth information using two deep network stacks. However, estimating 3D information from individual images is imprecise and creates uncertainty. To solve this problem, Karsch et al. [11] and Liu et al. [12] developed more in-depth learning methods and produced similar results using hand-crafted features such as the monocular depth estimation field. Furthermore, they combined data from multiple scales to improve the depth prediction at the pixel level [13]. Additionally, they used a conditional random field to greatly improve prediction rates [14]. Among recent studies, Zhou et al. [15] proposed a depth estimation method using an unsupervised learning algorithm to estimate depth information using an auto-encoder, which detects anomalies and converts them to depth information.

### 2.3. Depth Estimation Methods After GAN

**Figure 1:**
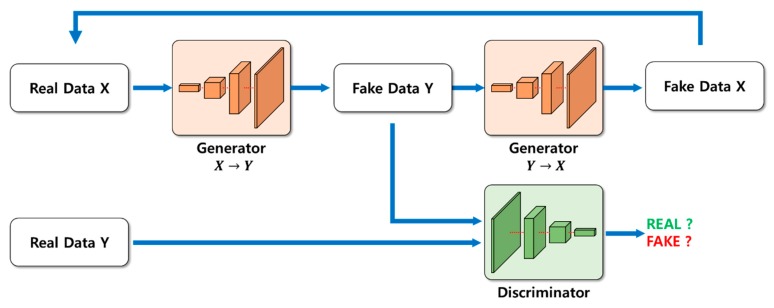
Structure of cycle generative adversarial network (GAN).

Meanwhile, a method of estimating depth information using GAN [16] has been proposed [17,18,19]. Afterward, the conditional GAN (cGAN) [20], which complemented the GAN, generated the desired data through the condition parameter by inserting it into the random noise, which was part of the generator input. The Pix2Pix [21], which transformed the cGAN, enabled the transformation of the input image into the desired image by replacing the existing random noise with an input image. Furthermore, using a generative neural network as a differential network, instead of fully loss-based learning, solved the underfitting and training pair problems [22,23]. However, the disadvantage was that a dataset pair was required to train the Pix2Pix. To solve the pair data problem, cycle GAN [24] added cycle consistency loss. However, there is still room for improvement when it comes to estimating depth information properly. The structure of the cycle GAN is shown in Figure 1. Considering the mapping problem for *X* dataset first, the output of the generator (*X* to *Y*) is likely to be virtually meaningless mapping, because it simply needs to look like the *Y* dataset. To address this, induce back to the original *X* dataset via the generator (*Y* to *X*). Thus, the generator will attempt to convert to *Y* dataset while retaining the form of *X* dataset, because it will have to perform two tasks of tricking the discriminator and returning to the original input data. Similarly, *Y* dataset performs the same process as above.

In this paper, cycle GAN was used to accurately estimate the depth information for the following reasons. First, it supports a higher resolution than other networks, even though the amount of learning data is large. Second, the shape of the depth information is consistent with the shape of the RGB image information. Shape is maintained simply means maintaining the form of RGB images and output images input into the neural network. Therefore, this does not mean that the resolution and number of channels are maintained. Moreover, by adding cycle consistency losses to the formula, the recovery of converted depth and segmentation information will result in the conversion to the original RGB image. This process was repeated to show the characteristic of fixing the output form of the generator. Therefore, it was determined that depth information would be useful in estimating it. For these reasons, we used cycle GAN to estimate depth information.

## 3. Depth Estimation Using Cycle GAN and Segmentation

**Figure 2:**
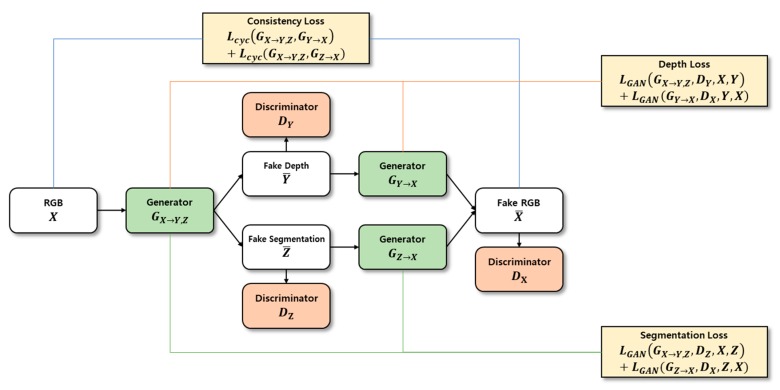
Outline of the proposed depth estimation method.

Our proposed depth estimation method using cycle GAN and segmentation is outlined in Figure 2. First, the depth information and segmentation images were created using a generator $G_{X\to Y,Z}$ for the input RGB image X and were restored as RGB images. Second, the estimated depth image, segmentation image, and restored RGB image were determined and compared with the learning dataset to calculate probability. Third, the resulting value was calculated using the cycle GAN’s objective function. Learning was adjusted so that the loss calculation during this process converged to zero, and the probability of the discriminator approached 50%. In addition, although the resulting values were different for the same input RGB image X owing to the cycle consistency loss, the original RGB image X and restored RGB image $\bar{X}$ were compared. This was accomplished as the image was converted back to RGB image $\bar{X}$ so that the depth image and segmentation image could be generated while still maintaining the shape of the original RGB image X. When learning was complete, the depth information was estimated using an existing generator $G_{X\to Y,Z}$.

**Figure 3:**
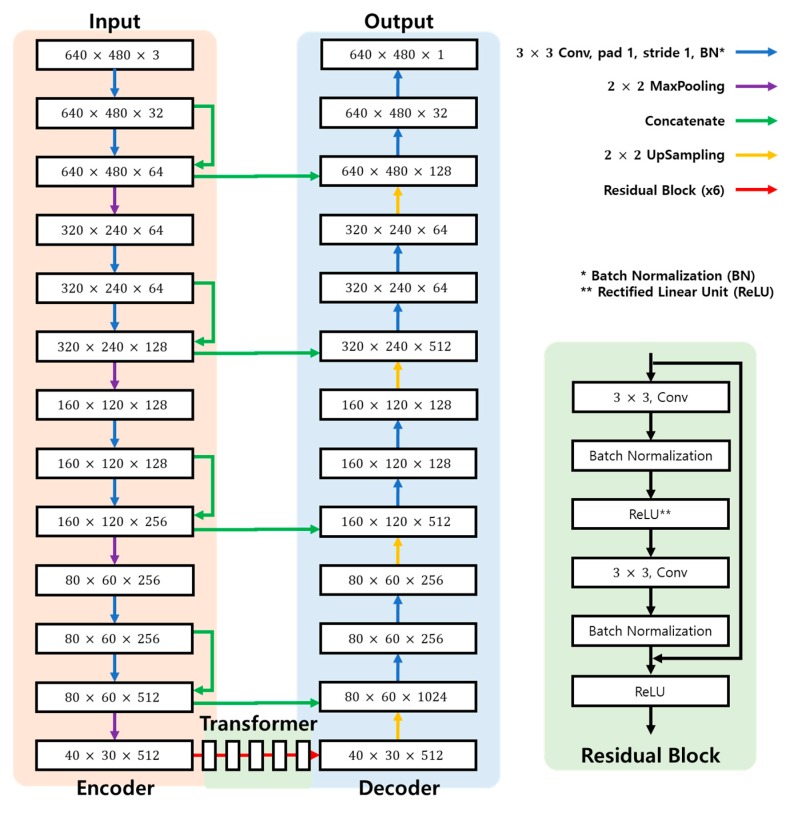
Structure of the generator applied in this study.

Figure 3 shows structure of the generator applied in this study. The network has 16 convolutional layers, and the filter size is 3 × 3. Generator’s encoder extracts feature for input image. Transformer transforms feature map generated by encoder into depth and segmentation feature using residual block. In the last decoder, a fake image of depth and segmentation is created.

**Figure 4:**
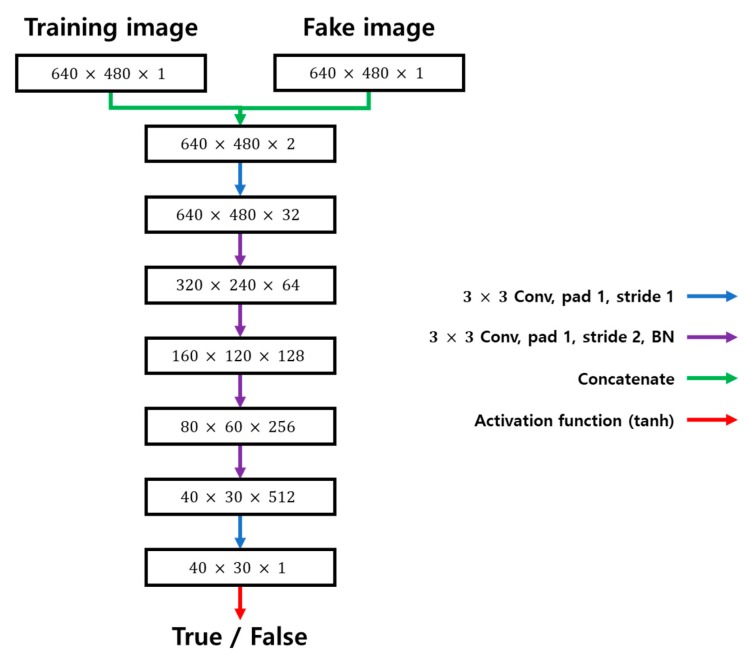
Structure of the discriminator applied in this study.

Figure 4 shows structure of the discriminator applied in this study. The network has six convolutional layers, and the filter size is 3 × 3. Tanh was used as an activation function. The input of the discriminator concatenates the training image with the fake image generated by the generator. The output of the discriminator shows the similarity between the data instance and the actual training dataset as a probability value between 0 and 1. The generated probability value is fed back to the generator again.

**Figure 5:**
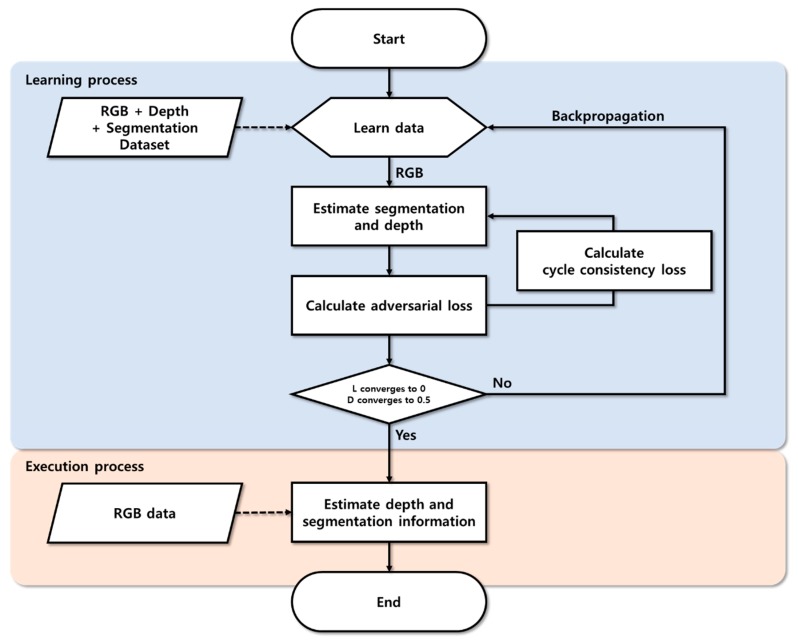
Flowchart for depth estimation method using cycle GAN.

Furthermore, our proposed method is composed of learning and inference processes, and the flowchart of the overall proposed method is shown in Figure 5. During the learning process, the segmentation and depth estimation methods were established. In addition, the learning algorithm was adjusted by calculating the objective function and discrimination probabilities. In the inference process, the depth information was estimated using only RGB images. The generator, which was used to learn depth information, was separated and used for depth estimation.

The reason that the proposed method can achieve higher resolution than other networks is because the ResNet [25] structure of the cycle GAN does not lose much information in high-resolution image processing. In ResNet’s residual block structure, the resolution can be maintained through a skip connection process, where existing computational results are combined. These structures were also applied to cycle GAN to maintain a relatively high resolution. Therefore, the proposed method was able to achieve a higher resolution than other networks.

### 3.1. Segmentation and Depth Estimation During the Learning Process

The operating sequence for the network is as follows. First, depth hints are provided through segmentation. To transform the RGB image information into segmentation information, the segmentation information is obtained by the generator, which is then used for depth estimation. In the same context, depth information for the RGB image is obtained by the generator that is used for semantic segmentation. The two generators that perform depth and semantic segmentation are fed back by the cycle consistency loss, which is later used to adjust the weights, and to determine the depth and semantic segmentation of the RGB image.

### 3.2. Calculation of Adversarial Loss During the Learning Process

The objective function of the network described in this paper is given by Equation (1), which is composed of the adversarial loss function of GAN and cycle consistency loss. Here, *X* represents RGB information, *Y* represents depth information, and *Z* represents segmentation information.

(1) $$\begin{array}{ll} L(G_{X\to Y,Z},\;G_{Y\to X}, & G_{Z\to X},D_{X},\;D_{Y},D_{Z})\\ & =L_{GAN}\left(G_{X\to Y,Z},\;D_{Y},\;X,\;Y\right)+L_{GAN}\left(G_{Y\to X},\;D_{X},Y,X\right)\\ & +L_{GAN}\left(G_{X\to Y,Z},D_{Z},X,Z\right)+L_{GAN}\left(G_{Z\to X},D_{X},Z,X\right)\\ & +\lambda_{1}L_{cyc}\left(G_{X\to Y,Z},G_{Y\to X}\right)+\lambda_{2}L_{cyc}\left(G_{X\to Y,Z},G_{Z\to X}\right) \end{array}$$

The adversarial loss function can be calculated by estimating the segmentation and depth information from the RGB image information, and the corresponding equations are shown in Equations (2) and (3). The core function of the adversarial loss function is to map the distribution generated from GAN to the actual distribution. Therefore, the adversarial loss function learns according to the Minimax results of the generator and discriminator. In an ideal case, the generator can generate a distribution that is analogous to the actual distribution, and the discrimination probability of the discriminator converges to 50%.

(2) $$L_{\mathrm{GAN}}\left(G_{X\to Y,Z},D_{Y},X,Y\right)=E_{y\sim P_{data}\left(y\right)}\left[logD_{Y}\left(y\right)\right]+E_{x\sim P_{data}\left(x\right)}\left[\log\left(1-D_{Y}\left(G_{X\to Y,Z}\left(x\right)\right)\right)\right]$$

(3) $$L_{\mathrm{GAN}}\left(G_{X\to Y,Z},D_{Z},X,Z\right)=E_{z\sim P_{data}\left(z\right)}\left[logD_{Z}\left(z\right)\right]+E_{x\sim P_{data}\left(x\right)}\left[\log\left(1-D_{Z}\left(G_{X\to Y,Z}\left(x\right)\right)\right)\right]$$

Furthermore, reconstruction loss is added to combine with the loss used in the existing CNN-based learning method. This is done so that the generative distribution of the generator can learn the standard distribution of the target. The reconstruction loss is expressed by the following:

(4) $$L_{\mathrm{Rec}}\left(G_{X\to Y,Z}\right)=E_{x\sim P_{data}\left(x\right),y\sim P_{data}\left(y\right),z\sim P_{data}\left(z\right)}\left[\|y-G_{X\to Y,Z}{\left(x\right)\|}_{1}\right]$$

### 3.3. Calculation of Cycle Consistency Loss During the Learning Process

Cycle consistency loss was configured to evaluate similarities between two estimated images, which were being restored from the original images separately using the two objective functions, as expressed by Equation (5). Cycle consistency loss is the sum of two losses, as this paper uses cycle GAN, which consists of two domains for segmentation and depth.

(5) $$\begin{array}{ll} L_{cyc}\left(G_{X\to Y,Z},G_{Y\to X}\right) & +L_{cyc}\left(G_{X\to Y,Z},G_{Z\to X}\right)\\ & =E_{x\sim P_{data}\left(x\right)}\left[\|G_{Y\to X}\left(G_{X\to Y,Z}\left(x\right)\right)-x{\|}_{1}\right]\\ & +E_{y\sim P_{data}\left(y\right)}\left[\|G_{X\to Y,Z}\left(G_{Y\to X}\left(y\right)\right)-y{\|}_{1}\right]\\ & +E_{x\sim P_{data}\left(x\right)}\left[\|G_{Z\to X}\left(G_{X\to Y,Z}\left(x\right)\right)-x{\|}_{1}\right]\\ & +E_{z\sim P_{data}\left(z\right)\;}\left[\|G_{X\to Y,Z}\left(G_{Z\to X}\left(z\right)\right)-z{\|}_{1}\right] \end{array}$$

**Figure 6:**
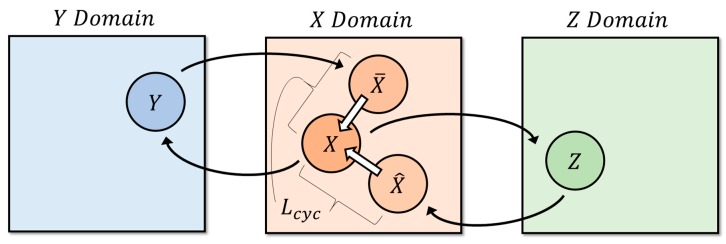
Cycle consistency loss $L_{cyc}$ for the cycle GAN model.

When examining cycle consistency loss, restoration of the generator must consider not only the restoration from the depth information, but also restoration from the segmentation information. Therefore, depth information using more object classification information can be generated by adding segmentation information to the existing method, which performs restoration only considering the depth information. By contrast, depth information is also considered when performing segmentation, which generates a synergistic effect for background separation. Figure 6 illustrates the cycle consistency loss.

Lastly, normalization of the cycle consistency loss was performed by imposing a penalty to the sum (L1 norm) of the absolute values for each element of the model with L1 loss weights. L1 loss is more robust than L2 loss, for an unstable solution problem. Therefore, L1 loss can be expressed as follows:

(6) $$d_{1}\left(p,q\right)=\|p-q{\|}_{1}=\overset{n}{\underset{i=1}{\sum }}\left|p_{i}-q_{i}\right|,\;\;\;where\;p=\left(p_{1},p_{2},\cdots ,p_{n}\right),\;q=\left(q_{1},q_{2},\cdots ,q_{n}\right)$$

In this paper, we induced the correlation between depth information and segmentation information through cycle consistency. Therefore, if you do not perform cycle consistency loss, you will not revert back to the RGB information from the computed depth information and segmentation information, which may result in poor output results. On the other hand, cycle consistency loss also has the effect of integrating depth information estimation and segmentation results. For example, a network’s generator that estimates depth information estimates depth information considering segmentation through cycle consistency loss.

### 3.4. Backpropagation

**Figure 7:**
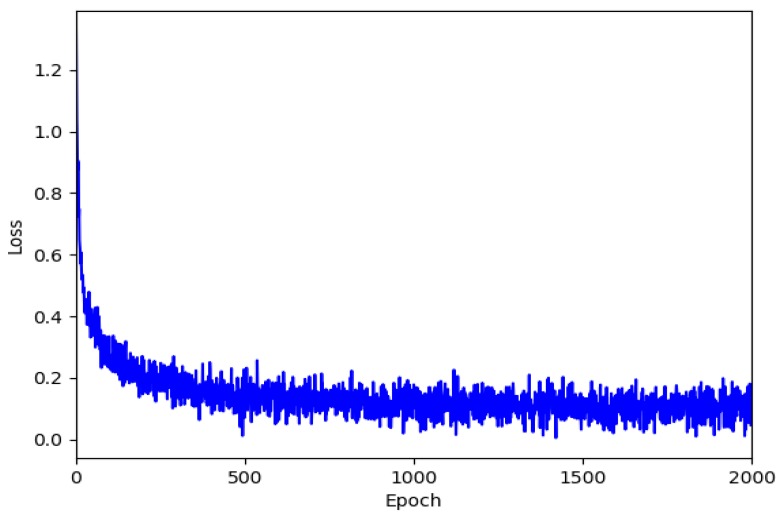
Loss value according to epoch for NYU Depth Dataset V2.

After calculating the adversarial and cycle consistency losses, the loss and discrimination probabilities for the generator and discriminator were calculated. The learning process was repeated so that the loss of the generator would converge to zero, and the discrimination probability of the discriminator would converge to 0.5. Figure 7 shows the loss of the repeated learning process for the NYU Depth Dataset V2. The system learned approximately 5000 images, while 200 test sets were used for validation. Meanwhile, the number of epochs was limited to approximately 2000, and only the smallest loss was selected.

### 3.5. Operating Sequence for the Learning Process 

**Figure 8:**
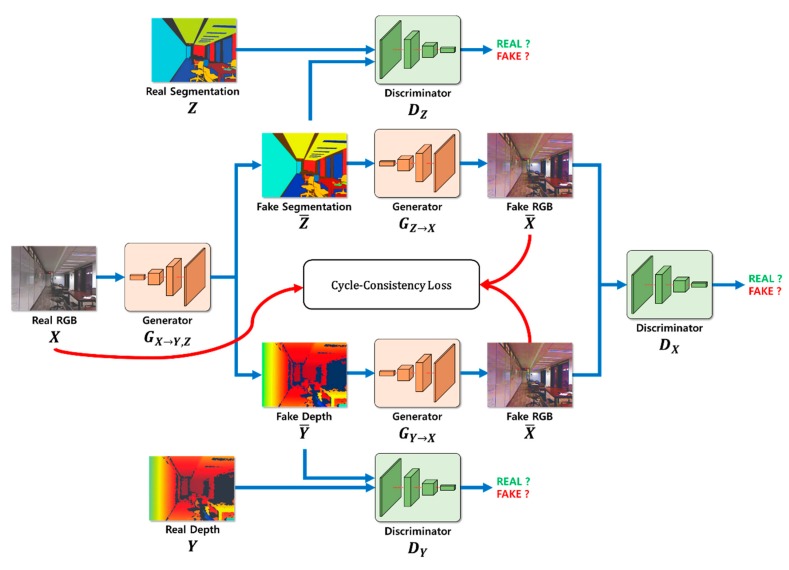
Operating sequences.

Figure 8 shows the operating sequences for estimating depth information by utilizing individual images using cycle GAN and segmentation. After the adversarial loss and cycle consistency loss were calculated, and the learning process was performed, the fake depth $\bar{Y}$ was finally estimated. As with the learning process, the sequence was divided into three processes: segmentation and depth estimation, adversarial loss calculation, and cycle consistency loss calculation.

#### 3.5.1. Segmentation and Depth Estimation Process

**Figure 9:**
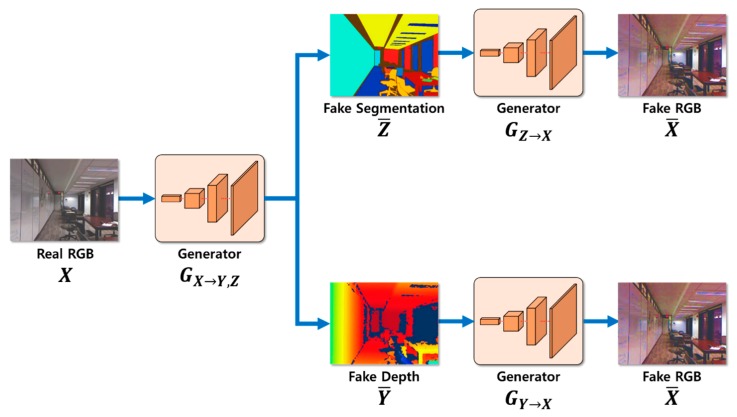
Segmentation and depth estimation process.

The output operation for the segmentation and depth estimation process from each generator is shown in Figure 9. The two networks have the same structure, and only the roles of the generator and discriminator are different. The operating sequence of the two networks is as follows. First, for an RGB input image X, segmentation and depth estimation were performed by generators $G_{X\to Y,Z}$. Afterward, the estimated images were restored again using generators $G_{X\to Y,Z}$.

#### 3.5.2. Adversarial Loss Calculation Process

**Figure 10:**
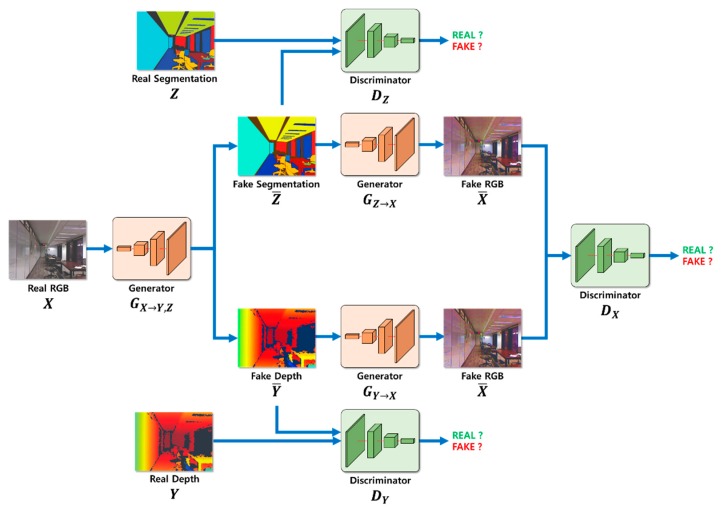
Adversarial loss calculation process.

The segmentation and depth estimated images were compared with the original images using the discriminator to calculate the probability. The adversarial loss was calculated using this process, as shown in Figure 10, and the calculated probability was fed back to the generator and discriminator through backpropagation.

#### 3.5.3. Cycle Consistency Loss Calculation Process

**Figure 11:**
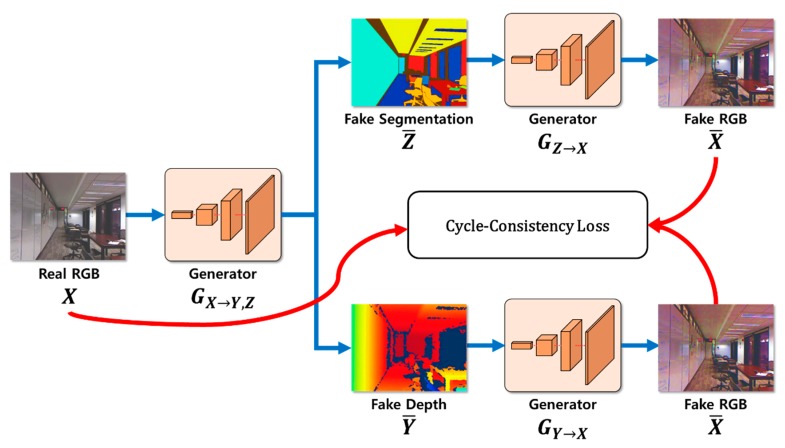
Cycle consistency loss calculation process.

The cycle consistency loss was calculated by comparing similar RGB images $\bar{X}$ restored by generators $G_{X\to Y,Z}$ with the original RGB image X. As shown in Figure 11, the cycle consistency loss was also fed back to the generator through backpropagation. The generator learned by considering the depth and semantic segmentation, adversarial loss, and RGB image restoration using cycle consistency loss.

### 3.6. Depth and Segmentation Information Estimation Inference Process

**Figure 12:**
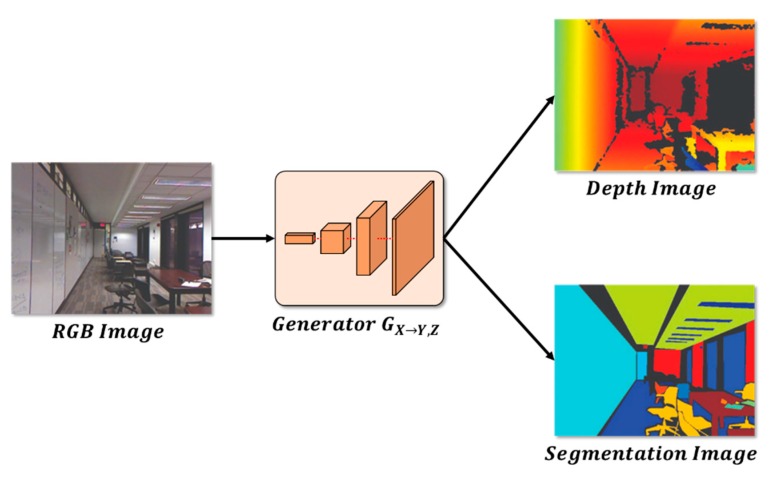
Network structure for estimating depth and segmentation information.

For the depth and segmentation information estimation method, the learning of cycle GAN completed when the estimation result was similar to the actual depth. Then, depth estimation was performed using a generator $G_{X\to Y,Z}$, which generated the depth image from the RGB image, like the actual depth and segmentation images, as shown in Figure 12.

Furthermore, semantic segmentation was performed using the same process. The RGB image was transformed into segmentation information using generator $G_{X\to Y,Z}$, which was used for the learning process.

## 4. Results and Discussion

### 4.1. NYU Depth Dataset V2 Result

**Figure 13:**
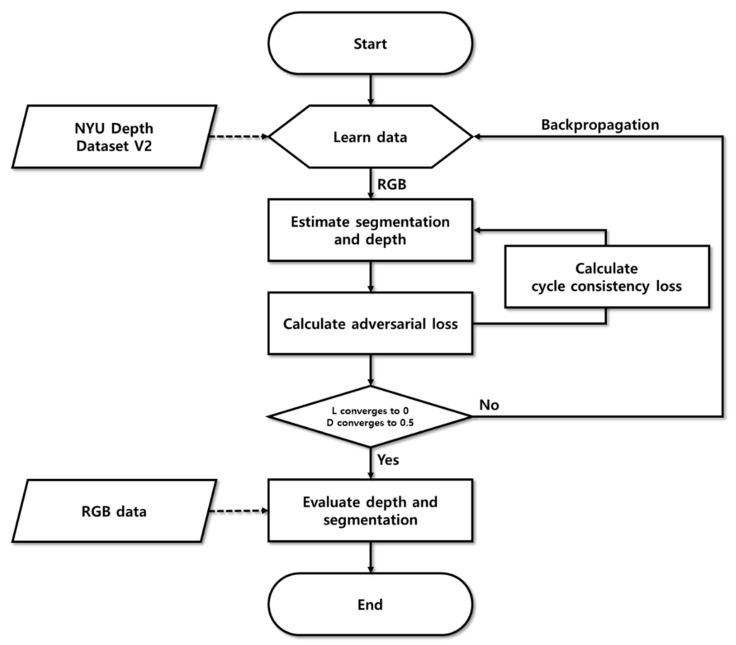
Experimental process.

To evaluate the reliability of the proposed depth information estimation method using cycle GAN and segmentation, an experiment was conducted using NYU Depth Dataset V2, an open-source database. The experimental process for the proposed method, which estimated depth information from individual images using cycle GAN and segmentation, is shown in Figure 13. In the depth and semantic segmentation step, the learning rate was calculated using the objective function, while the depth and segmentation information was estimated. The effects of the combined cycle consistency losses for each domain are proposed in this paper. In the GAN objective function calculation step, only the adversarial losses were calculated from the previous depth and semantic segmentation step. As there was no intersection between depth and segmentation in this step, the depth and segmentation were estimated independently. In the cycle consistency loss step, depth and segmentation images were generated in the depth and semantic segmentation step and were converted into original RGB images. After calculating the cycle consistency loss by comparing the restored RGB image with the original RGB image, a penalty was imposed on each generator of depth and segmentation. Throughout this process, the generator performed depth and semantic segmentation by considering the restoration of depth and segmentation.

**Figure 14:**
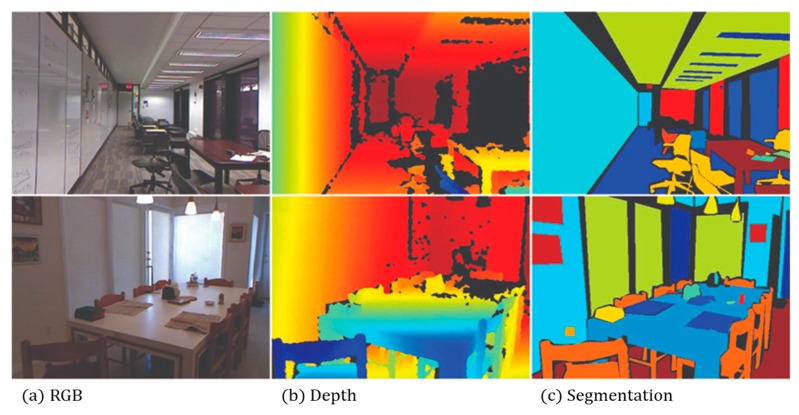
NYU Depth Dataset V2.

To evaluate the proposed objective performance of the depth estimation method using cycle GAN and segmentation, the NYU Depth Dataset V2 [26] database was used. The NYU Depth Dataset V2 database provides video sequence data for various indoor scenes using Microsoft’s Kinect v1 model, as shown in Figure 14. In addition, depth and segmentation information for the RGB images was provided through labeled datasets. For this study, we used approximately 5000 learning images. 

The hardware used for this experiment was composed of a system with an Intel(R) Core(TM) i7-8700 K 3.70 GHz CPU processor with 16 GB of RAM, an NVIDIA GeForce GTX 1080 Ti (V-RAM 11 GB) GPU, and a Windows 10 Pro 64 bit operating system. For software development, Visual Studio version 2017 was used. For the libraries, we utilized OpenCV2, CUDA 10.2, and cuDNN v7.6.5. The deep learning library was Pytorch version 1.3.

**Figure 15:**
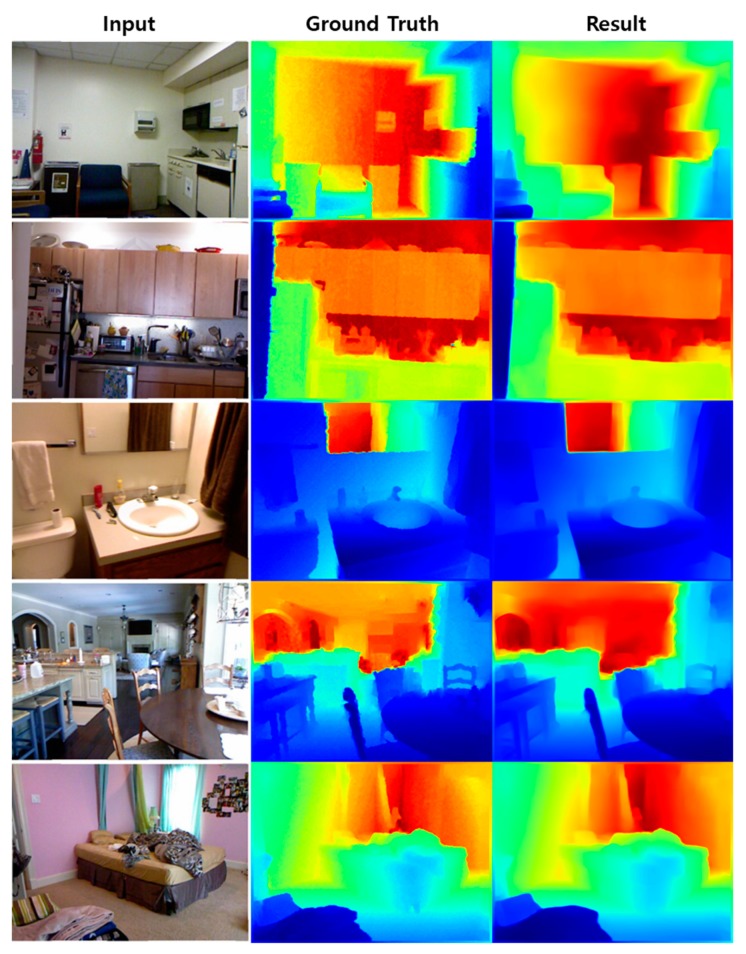
Depth estimation results of the images of NYU Depth Dataset V2.

In this study, we estimated the depth using approximately 200 test sets in addition to the training sets used for learning. The results are shown in Figure 15.

Table 1 compares the proposed method with the other methods published in other papers using the NYU Depth Dataset V2 to evaluate the objective reliability. In Table 1, the depth estimation technique based on Zheng et al. [19] GAN was used, while Eigen et al. [10] and Liu et al. [12] used the depth estimation technique based on CNN. The lower Root Mean Square Error (RMSE) and Root Mean Squared Logarithmic Error (RMSLE) values indicate a better depth estimation method. The higher $\delta_{1}$, $\delta_{2}$, and $\delta_{3}$ values indicate a better depth estimation method. Therefore, our proposed method is more efficient, as it has exhibits better depth estimation than other methods. The papers in Eigen et al. [10] and Liu et al. [12] did not show the RMSLE values, so we did not display them in Table 1.

Table 2 compares the depth estimation result that removed segmentation with the depth estimation result that combined depth and segmentation. From Table 2, it can be seen that the result of combining depth and segmentation was superior to the result of removing segmentation. Thus, the effect of depth estimation combined with depth and segmentation was proven.

### 4.2. Custom Result

**Figure 16:**
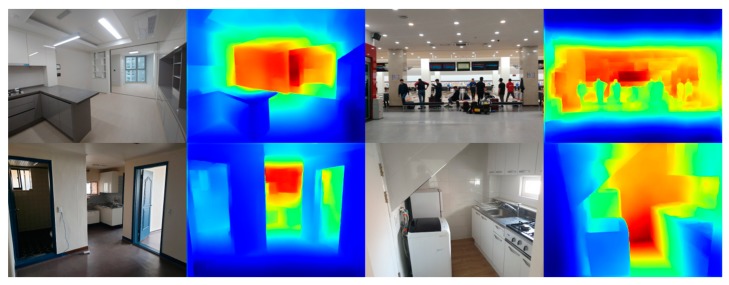
Result of proposed method for custom photos.

The results of the depth estimation by taking pictures of various landscapes with a smartphone camera are as follows. To measure values, we must have actual depth information for that image. However, it was not possible to compare the actual depth information from the video taken through a smartphone camera. Figure 16 is the result of performing a depth estimation through the program and determines that the depth estimation is adjudged to have been accurately carried out.

## 5. Conclusions

This paper proposes a depth estimation method using cycle GAN and segmentation. This contrasts with existing depth estimation methods that require special equipment and multiple images. Cycle GAN was used because although the amount of data required for learning was large, the system supports higher resolutions than other networks, and RGB image information was not changed. To estimate the depth information, our proposed method only used single RGB images, and it could easily transform the images using cycle GAN, even with an unpaired dataset. Furthermore, the image information was transformed to depth information while maintaining the characteristics of the RGB image, owing to the consistency loss of cycle GAN. In addition, the loss of depth information was minimized by adding the consistency loss of segmentation. The fading problem where depth information is ambiguous or hidden by larger features was also solved. To evaluate the objective reliability of the proposed method, it was compared with other methods using the NYU Depth Dataset V2. Our results show that the benchmark value for our proposed method is better than other methods. This shows that, since the introduction of deep learning techniques [27], it has become very difficult to estimate depth, a measure of MDE. Therefore, we demonstrated that our proposed method is more efficient in determining depth estimation. In the future, various types of information should be raised to the level of actual measurement data through the synergistic effect of multiple domains using various types of information, as well as through segmentation by utilizing the scalability of cycle GAN.

Further research is required to apply the depth information generated in this study to AR and 3D vision fields using 3D maps and objects.

## Figures and Tables

**Table 1:** Comparison of our method to existing methods on NYU Depth Dataset V2.

Method	RMSE↓	RMSLE↓	δ_1_↑	δ_2_↑	δ_3_↑
Zheng et al. [19]	0.915	0.305	0.540	0.832	0.948
Eigen et al. [10]	0.909	-	0.602	0.879	0.970
Liu et al. [12]	0.824	-	0.615	0.883	0.971
The Proposed Method	0.652	0.217	0.834	0.941	0.976

**Table 2:** Comparison of our method with depth only method using NYU Depth Dataset V2.

Method	RMSE↓	RMSLE↓	δ_1_↑	δ_2_↑	δ_3_↑
Depth only	0.687	0.228	0.819	0.936	0.972
The Proposed Method	0.652	0.217	0.834	0.941	0.976
